# Influencing factors on the tendency of general practitioners to join in urban family physician program: a cross-sectional survey on Iranian physicians

**DOI:** 10.1186/s12962-022-00373-0

**Published:** 2022-08-17

**Authors:** Mohsen Bayati, Arash Rashidian, Vahid Yazdi-Feyzabadi, Sajad Delavari

**Affiliations:** 1 Health Human Resources Research Center, School of Health Management and Information Sciences, Shiraz University of Medical Sciences, Shiraz, Iran; 2 Department of Science, Information and Dissemination, Eastern Mediterranean Regional Office, World Health Organization, Cairo, Egypt; 3 Health Services Management Research Center, Institute for Futures Studies in Health, Kerman University of Medical Sciences, Kerman, Iran

**Keywords:** Family physician, General practitioners (GPs), Personal satisfaction, Income, Primary healthcare

## Abstract

**Background:**

Urban family physician program (UFPP) is initiated as pilot by policy makers as a main reform in future of primary health care in Iran. Despite an ongoing pilot implementation of this program from 2012, it remains a main question about providing sufficient number of general practitioners (GPs). This study aimed to investigate the factors which affect GPs’ decision to join in the UFPP.

**Methods:**

In this national cross-sectional study a sample of 666 GPs, using convenience sampling, filled a self-report questionnaire. The multivariate logistic regression was applied to explore the demographic, practice and views determinants of the tendency of GPs to join in the UFPP.

**Results:**

More than half of GPs (58.6%) participated in the study had a positive tendency to join in the UFPP. Older GPs (adjusted OR = 3.72; 95%CI 1.05–13.09), working in public sector (adjusted OR = 2.26; 95%CI 1.43–3.58), lower income level (adjusted OR = 6.69; 95%CI 2.95–15.16), higher economic expectations (adjusted OR = 2.08; 95%CI 1.19–3.63), and higher satisfaction from medicine profession (adjusted OR = 2.00; 95%CI 1.14–3.51) were the main factors which increased the GPs tendency to enter in UFPP.

**Conclusions:**

Decision for joining in the program is mainly affected by GPs’ economic status. This clarifies that if the program can make them closer to their target income, they would be more likely to decide for joining in the program.

## Introduction

Iranian health system has achieved many improvements in health outcomes following the establishment of health network system based on primary health care [1]. Herein, these achievements and establishing rural family physician has made Iran as one of good practices across the world. However, despite these merits it also suffers from some drawbacks such as ineffective referral system in urban areas [2]. As, many experts and policy makers for many years are exploring and working on designing an effective system in which the primary health care (PHC) in urban areas work as much as rural areas. On this basis, establishing family physician program has proposed for some years as most important solution to this inefficient system in urban areas [3, 4].

Family physician program with the primary goal of implementing complete referral system and accessibility of population to needed health services which started in 2005. It started from rural areas which has had several achievements as well as deficiencies [5]. One of the main achievements is increase in number of general practitioners (GPs) in rural areas and as a result accessibility of nearly all rural population to GP services [6]. On the other hand, GPs were not educated for working in the plan and this results in some problems [6, 7]. Besides, GPs are dissatisfied with the program [8] as well as financing challenges regarding to the program [6].

The program was highly emphasized in upstream documents and health policy makers were committed to expand it. In this regard, form 2012–13 two provinces in the country including Fars and Mazanderan were formally selected as settings for pilot implementation of urban family physician program (UFPP) in Iran. This program aims to respond many challenges such as high out of pocket payments for medical services, better medical and clinical pathways, unnecessary straight referrals to specialists, and more effective accessibility through reallocating the current health resources to more necessary and complex health services [9].

According to this program, the same as rural family physician program, GPs should play the pivotal role as a gatekeeper for providing and managing necessary health services in first contact [9]. At first, the GPs should enroll in the program and thereby covered a defined number of people mostly based on a geographical catchment area. The GPs, as head of health teams, are responsible to meet health needs of population as well other members such as health worker, nurse, midwife, nutritionist, and psychologist. They should also play a role as gatekeeper to meet the medical needs and guiding to higher levels (i.e. specialists and other providers with more complex services) through establishing an effective referral system [9].

The UFPP is now continuing as pilot in both mentioned provinces [5]. Based on emphases of policy makers and upstream documents, it will be a fundamental program in future. As well, due to successful experiences of family physician program, many countries are planning to initiate or develop it. Though, it needs studies to predict its feasibility and outcomes.

One of main concerns in each health policy and generally achieving to universal health coverage is providing and equitable distribution of efficient and effective human resources [10, 11]. Furthermore, each improvement in health care services is highly depended on health workforce that is fit for the purpose [11]. Thus, identifying and addressing to the factors affecting join in human resources in the policies is an inevitable effort. In case of UFPP in Iran, this issue is more important. Since, two-third of people in Iran are resident in urban areas and to some extent we are face with shortage of GPs that is calculated about 36% in a recent study [12]. As well, Iran urban settings in contrast to rural areas have some structural characteristics which may hinder the implementation of family physician in urban settings. In fact, the passive health network, powerful private sector which have conflict of interest with UFPP, population freedom for choosing health provider, and different cultural norms are those factors that make UFPP more different from rural family physician program [4].

Some studies conducted in different countries shows that diverse push factors within and outside health system might affect the tendency of human resources to join in the policies [7, 13]. Results of these studies are also different which clarifies the factors are multi-facetted and complex [7, 14] and are mainly categorized into three groups including contextual/environmental, personal and professional factors [13]. In this regard the approach to the study might be different from one study to another study. Some studies have conducted on the human resources which were experiencing the job and some other were focused on the workforce prior to joint in the program. For example, a previous study conducted on factors affecting leave out the GPs in Iranian rural family physician showed that opportunity for continuing education, inappropriate and long working hours, unsuitable requirements of salary, irregular payments, lack of job security and high working responsibility were regarded as the most important reasons for leaving out the program in the past and intention to leave out in future [15].

Notwithstanding some studies were conducted on this area, we could not find any research to study factors affecting joining the GPs in the Iran’s UFPP. Furthermore, it is necessary to know the influencing factors prior the extension of this program to whole country. It might help the policy makers to make more effective decisions to advance the program in the future. Therefore, this study aimed to explore the tendency of GPs to join in the UFPP and find the factors might effect on it.

## Methods

### Study sampling

A cross-sectional study was used for conducting this study. The research population were Iranian GPs. Several methods were considered for sampling all GPs across the country. Medical Council of Iran (MCI), the most prominent societies in medical field, register all practitioners’ information which could be a sampling frame. Another database is the white book which published physician information. But after several inquiries it was found that this two sampling frames are out-of-date. As well, a study about accuracy of Iranian physician directories report their accuracy less than 60% [16]. Therefore, physician directories were assumed unreliable. Another way was sampling the congresses which are held by the Iranian Society of General Practitioners (ISGP). The ISGP holds main congresses as re-training programs for Iranian GPs that obliges all GPs to attend them. Though, this congresses are assumed to be a representative sample of all GPs while all GPs across the country attend them. Knowing the limitation of this sample, summer and autumn national congresses in 2015, which about 3500 GPs enrolled, were selected for sampling. The questionnaires were distributed among 1209 GPs during break times that 666 questionnaires were returned (55.1% response rate). The inclusion criteria for participants was being a GP, and having active clinical practice. If the participants were student in higher levels were excluded from the study.

### Study variables

According to aim of the study and literature review, the primary model was presented as follows:

Tendency to join in UFPP=f (demographics, practice, and views/perspectives)

The variables of model were selected based on data availability. Therefore, age, gender and marital status as demographic factors; experience, location, type and actual income from practice as practice factors; and perceived socio-economic status (SES), economic expectations and satisfaction from medicine profession as views/perspectives factors were included in the primary model.

The data were collected using a valid and reliable questionnaire which was developed and published by the authors elsewhere [17, 18]. The views/perspectives variables were obtained by one or more questions as follows:

### Perceived SES

This included two items: “economic status of the population are living at neighborhood of GP home place” and “economic status of the population are living at neighborhood of GP practice place”.

### Economic expectations

This consists of three items: “economic expectation of the own GP”, “economic expectation of GP’s family” and “economic expectation of society”.

### Satisfaction with medicine profession

This variable was asked via the following question:

If you return back, would you choose medicine again?" the responses were categorized into yes, somewhat, and no.

The scores of compound variables (perceived SES and economic expectations) were estimated using the principal component analysis (PCA) technique.

### Data analysis

Since rural GPs mostly practice in rural family physician program, we limited statistical analysis to GPs were working in urban areas. Furthermore, the questionnaires with non-response for main variables were excluded from the analysis. Descriptive statistics and logistic regression model were used for data analysis. The study variables were simultaneously (enter method regression) entered into the final model using logistic regression analysis. STATA software version 14.0.

### Ethical consideration

The study follows several ethical considerations including confidentiality of questionnaires and free will to participate in the study. As well the study protocol was approved by ethics committee of Shiraz University of Medical Sciences (with registration code of IR.SUMS.REC.1397.794) and complies with the ethical guidelines of the 1975 Declaration of Helsinki.

## Results

The response rate of questionnaire was 55.1%. From all 666 returned questionnaires distributed, 601 GPs (90.2% of participants) were working in urban areas were returned (52.6% of all distributed questionnaires). The descriptive statistics of the variables are shown in Table 1. About half (n=330; 54.9%) of GPs were male and more than two third (n=410; 68.2%) of them were placed in two middle age groups (36–45 and 46–55 years). Most of them (n=498; 82.9%) were married. More than half of GPs (n=352; 58.6%) had tendency to enter in UFPP in the future.

**Table 1:** Definition and descriptive statistics of the variables (n = 601)

Variable	Frequency (%)	Have tendency to join UFPP (%)
Gender		
Male	330 (54.9)	117 (39.4)
Female	271 (45.1)	105 (43.4)
Age groups		
26–35	126 (21.0)	38 (36.2)
36–45	189 (31.4)	64 (37.2)
46–55	221 (36.8)	87 (44.6)
≥ 56	65 (10.8)	29 (52.7)
Marital status		
Single	103 (17.1)	32 (36.8)
Married	498 (82.9)	190 (42.2)
Practice experience		
0–5	141 (23.5)	48 (39.7)
6–10	94 (15.6)	33 (41.8)
11–15	123 (20.5)	49 (42.6)
16–20	142 (23.6)	48 (37.8)
≥ 21	101 (16.8)	41 (48.2)
Practice location		
Tehran (Capital of Iran)	245 (40.8)	92 (45.1)
Other province centers	167 (27.8)	57 (41.60
Other cities	189 (31.4)	49 (33.6)
Practice setting		
Private	371 (61.7)	125 (36.7)
Public	230 (38.3)	97 (49.0)
Income quintile		
Poorest	141 (23.5)	68 (56.7)
Poor	116 (19.3)	50 (49.0)
Middle	112 (18.6)	38 (39.6)
Rich	110 (18.3)	33 (34.4)
Richest	122 (20.3)	24 (24.5)
Perceived SES		
Low	205 (34.1)	81 (45.5)
Medium	227 (37.8)	81 (40.5)
High	169 (28.1)	59 (38.8)
Economic expectations		
Low	204 (34.0)	64 (37.0)
Medium	213 (35.4)	75 (40.1)
High	184 (30.6)	75 (47.8)
Satisfaction with medicine profession		
Low	134 (22.3)	46 (38.7)
Medium	129 (21.5)	43 (37.7)
High	338 (56.2)	130 (44.8)
Total	601	325 (58.6)

Finding also showed that GPs in terms of “practice experience”, “practice location” and “income quintile” variables, were distributed almost equally among defined different classes. About two third (n=371; 61.7%) of GPs worked in private sector such as private offices, clinics and hospitals. Moreover, views/perspectives variables including “perceived SES” and “economic expectations”, GPs were about equally distributed in low, medium and high groups. However, about “satisfaction from medicine profession”, more than half (n=338; 56.2%) of GPs were highly satisfied from their profession and others had a low to medium satisfaction.

The analytic results from logistic regression analysis are shown in Table 2. Gender (adjusted OR=0.80; 95% CI 0.47-1.37) and marital status (adjusted OR=1.20; 95% CI 0.63–2.29) did not show significant relationship with tendency to enter in the UFPP. However, GPs with higher age had a more tendency to join in the UFPP (adjusted OR=3.72; 95% CI 1.05–13.09).

**Table 2:** Determinants of GPs’ tendency to join in urban family physician program (n = 601)

Variable	$\beta$ Coefficient	Adjusted OR	95% CI
Gender			
Male	Reference	–	–
Female	− 0.21	0.80	0.47–1.37
Age group			
26–35	Reference	–	–
36–45	0.45	1.57	0.68–3.60
46–55	1.21	3.36	1.29–8.70^*^
≥ 56	1.31	3.72	1.05–13.09^*^
Marital status			
Single	Reference	–	–
Married	0.18	1.20	0.63–2.29
Practice experience			
0–5	Reference		
6–10	− 0.33		
11–15	− 0.45	0.63	0.30–1.66
16–20	− 0.96	0.38	0.26–1.53
Practice location			
Tehran (Capital of Iran)	0.42	1.53	0.87–2.67
Other province centers	0.17	1.19	0.66–2.12
Other cities	Reference		–
Practice setting			
Private	Reference	–	–
Public	0.81	2.26	1.43–3.58^*^
Income quintile			
Poorest	1.90	6.69	2.95–15.16^*^
Poor	1.34	3.84	1.74–8.46^*^
Middle	0.74	2.11	1.01–4.44^*^
Rich	0.67	1.95	0.92–4.12^**^
Richest	Reference	–	–
Perceived SES			
Low	Reference	–	–
Medium	− 0.39	0.67	0.39–1.15
High	− 0.52	0.59	0.32–1.01^**^
Economic expectations			
Low	Reference	–	–
Medium	0.38	1.46	0.85–2.50
High	0.73	2.08	1.19–3.63^*^
Satisfaction from medicine profession			
Low	Reference	–	–
Medium	0.05	1.05	0.54–2.03
High	0.69	2.00	1.14–3.51^*^
Constant	− 2.61	0.07	0.01–0.37

LR chi^2^ = 62.13; P = 0.0001

^*^Pseudo R^2^ = 0.11

^*^Statistically significant at *P* < .05

^**^Statistically significant at *P* < .10

*SES* socio-economic status, *CI* confidence interval, *OR* odds ratio

There was no significant relationship between practice experience (adjusted OR=0.44; 95% CI 0.14–1.34) and practice location (adjusted OR=1.53; 95% CI 0.87–2.67) with the decision of GP to enter in the UFPP. GPs who worked in public sector had a higher probability (more than 2 times) to join in the UFPP as compared with GPs in private sector (adjusted OR=2.26; 95% CI 1.43–3.58). GPs with lower actual income level had significantly more tendency to participate in the UFPP; so that GPs with lowest income (poorest quintile) had more than 6 times probability to participate in UFPP as compared to GPs with highest income quintile (adjusted OR=6.69; 95% CI 2.95–15.16).

GPs with higher level of perceived SES had lower tendency to enter in UFPP but with a low significance level (p<0.10). It was found that there is a significant relationship between GPs with higher economic expectations and more tendency to enter them in the UFPP (adjusted OR=2.08; 95% CI 1.19–3.63). Furthermore, GPs with high satisfaction form their profession had a more preference to join in the UFPP (adjusted OR=2.00; 95% CI 1.14–3.51).

## Discussion

This study was undertaken to identify the influencing factors on decision of GPs to join in UFPP in Iran. Since Iran is planning to develop UFPP to the whole country and maybe some other countries plan to implement it, identifying the factors affecting on join the GPs in the program is necessary. This issue is even more important where the ratio of GPs numbers to urban population is faced with a remarkable shortage. Thus, the results of this study provide evidence for policy makers to adopt suitable strategies and policies to overcome the barriers to join in the program.

This study has identified that higher age, working in public settings, lower self-reported income level, and lower level of perceived SES status, higher economic expectations, and satisfaction from the medicine major in GPs were the main factors that increase the probability to join in the UFPP. Although these factors might grouped into push factors including demographics, practice and view/perspective-dependent factors within and outside in the health sector separately, it is necessary to note that they might also interact with and influence each other [14].

The current study found that older GPs might have more tendency to join in the UFPP. In this regard, it seems that age is a personal factor which its impact is fluid and might be changed highly in matching with the career cycle [19, 20]. It seems that older GPs have more family responsibilities [13] and they might be more risk averse since that they want to have more stable conditions professionally. The family physician program in Iran compensate their services on a basis of per capita payment system [3, 21]. Thus, this may financially undergo less risk for the GPs with higher age.

Another result was that the GPs who are working in public sector (public clinics or hospitals) have more tendency to join in the UFPP as compared to those in private settings. This issue might be as a result of some reasons. First, GPs employed in public sector attain most of their income from outpatient visits and consultations based on public tariffs or fixed salary [3]. So they prefer to practice as family physician which is similar to their current practice mechanism (visit based) but with a per capita payment system based on coverage of a defined population. This issue may increase their income level. Second, GPs employed in private sector almost practice without public sector limitations. In other words, they behave and practice freely than GPs in public settings. So GPs employed in private settings have less tendency to join in the program with some limitations. Nonetheless, it is necessary to be conducted further research for identifying the differences between the public and private settings which might affect the tendency to enter in the program.

The perceived SES and self-reported income level of the GPs might also highly affect the tendency to enter in the program. It seems that income level of each person has a direct relationship with her/his selected job. This issue is even more important in health care industry considering that the inflation rate in this sector is higher than other general sectors in the society [22]. So, the less income and lower SES, the more tendency to join in the program will be. A recent study found that GPs are not satisfied with UFPP as it decreases their self-esteem and social status [23]. The UFPP provide a stable and medium level of earning to GPs, therefore GPs with low income/low SES tend more to participate in the program. This finding is consistent with other studies conducted in health sector that income level has a considerable effect on the type of job that each worker in health sector might select [22, 24–26].

One interesting finding is that GPs who are satisfied with their medicine profession, have a tendency more to enter in the program. This issue clearly indicates that job intrinsic motivations which are mainly intangible might to some degree be effective in joining in the program. It might also be noted that most likely the UFPP is compatible with the job intrinsic values in the medicine profession. This program provides serving to the community through more contact with the people. Also it is asserted by some authors that satisfaction from medicine profession can effect on physician’ economic behaviors and decisions such as retention, target income, turnover and productivity [27, 28].

The most interesting finding was that GPs with higher economic expectations might also increase the preference to enter in the UFPP. This issue can be explained by target income hypothesis [29]. According to this theory, whatever the gap between actual and target income for GPs be more, they seeks the ways to be enabled them to decrease the gap through attaining more income [30–33]. So it seems UFPP might close the GPs to their target income or conform to their economic expectations. This emphasizes the importance and mix of the revenue generating activities in the health policies.

A key strength of our study was the variables explored are a relatively comprehensive set of potential determinants which may affect the decision to enter in the program especially those related to GPs’ perspectives such as satisfaction and affection with the medicine profession and economic expectations. Since, as theoretically clarified in the literature, the decisions and behaviors of GPs are affected by the preferences and perspectives of them [29].

This study also had three main limitations. First, the findings may be somewhat affect by the sampling method. The participants (i.e. GPs) were sampled from a national educational congress conducted for GPs which many GPs presented from all of the country; however, the sampling method was as haphazardly. This so may limit the generalizability of the study which it necessitates so we cautiously interpret the results. So, we suggest further research to be conducted for more precise results. Second was the relative low response rate which was 55.1%. Third, the data produced were self-report; therefore, it maybe makes the measurement bias. However, it should be also noted that this is common in individual or household surveys [34].

In summary, this study is one of the first studies conducted to determine the influencing factors on the tendency the GPs to enter in the UFPP. As it explicitly is emphasized in the upstream documents, the establishment of referral system relied on primary health care and family physician for cities more than 20000 populations are a cornerstone in the future of Iranian health system. This study provided some preliminary evidence for identifying the factors affecting the GPs to tend for joining in the program. Furthermore, it also is necessary to be conducted further research with more depth and more rigorous methodology.

## Conclusion

Overall, this study indicated that practice related factors such as income are one of most drivers for effective implementation of the program. Since the Iranian health system is faced with a shortage of GPs in the program, should address more the factors that might affect the joining and retention of GPs. This study also in light of target income theory indicated that decision to join in the program by GPs is highly depended on their economic views especially economic expectations. This issue clarifies that whatever the program can close them to their target income, most likely they will decide for joining in the program. Finally, it seems that government can have much influence on both the local and national environment in the health sector for applying efficient strategies to encourage for joining in the program.

## Data Availability

The datasets gathered and analyzed during the current study are available from the corresponding author on reasonable request.

## Abbreviations

GP: General practitioner
UFPP: Urban family physician program
MCI: Medical Council of Iran
ISGP: Iranian Society of General Practitioners
SES: Socio-Economic Status
PCA: Principal component analysis
